# Metabolic Syndrome and Migraine

**DOI:** 10.3389/fneur.2012.00161

**Published:** 2012-11-19

**Authors:** Amit Sachdev, Michael J. Marmura

**Affiliations:** ^1^Department of Neurology, Jefferson Headache Center, Thomas Jefferson UniversityPhiladelphia, PA, USA

**Keywords:** migraine, metabolic syndrome, obesity, hypertension, hyperlipidemia, insulin resistance, prothrombotic state, inflammatory state

## Abstract

Migraine and metabolic syndrome are highly prevalent and costly conditions. The two conditions coexist, but it is unclear what relationship may exist between the two processes. Metabolic syndrome involves a number of findings, including insulin resistance, systemic hypertension, obesity, a proinflammatory state, and a prothrombotic state. Only one study addresses migraine in metabolic syndrome, finding significant differences in the presentation of metabolic syndrome in migraineurs. However, controversy exists regarding the contribution of each individual risk factor to migraine pathogenesis and prevalence. It is unclear what treatment implications, if any, exist as a result of the concomitant diagnosis of migraine and metabolic syndrome. The cornerstone of migraine and metabolic syndrome treatments is prevention, relying heavily on diet modification, sleep hygiene, medication use, and exercise.

## Introduction

Migraine is a condition that is usually characterized by a moderate or severe throbbing headache, unilateral pain, nausea or vomiting, light and sound sensitivity, and disability. Neurologic, gastrointestinal (Aurora et al., 2007), and autonomic symptoms (Blau, 1992) may be present. Experts believe that some individuals have a genetic predisposition for migraine (Van Den Maagdenberg et al., 2010) and defective pain control mechanisms (Aderjan et al., 2010). Vascular dilation and inflammation (Viola et al., 2010) occur in acute migraine, but migraine is now felt to be a neuronal disorder (Ba’albaki and Rapoport, 2008). Migraine pathogenesis involves the release of inflammatory neuropeptides [such as calcitonin gene-related peptide (CGRP)], trigeminal activation (Buzzi and Tassorelli, 2010), and cortical spreading depression (Milner, 1958; Ayata, 2010; Viola et al., 2010). Migraine is associated with many comorbid central and peripheral disorders, such as ischemic stroke, Raynaud phenomenon, epilepsy, depression, anxiety, obesity, and possibly metabolic syndrome (Guldiken et al., 2009; Buse et al., 2010). The interplay and causality of migraine and its comorbid disorders is not well understood (Marmura, 2009), particularly in regard to migraine’s association with obesity and metabolic syndrome.

Metabolic syndrome includes a number of abnormalities, including hypertension, insulin resistance, atherogenic dyslipidemia, a proinflammatory state, a prothrombotic state, and increased waist circumference or obesity (Alberti et al., 2006). Several organizations have proposed diagnostic criteria for metabolic syndrome (Alberti et al., 2006), contributing to confusion concerning the most useful definition in any given patient population. Some have questioned if diagnosing metabolic syndrome is useful to patient care (Kahn et al., 2005; Anscombe et al., 2006). The presence of metabolic syndrome has been associated with a significant risk of cardiovascular disease (Mottillo et al., 2010), stroke (Gupta et al., 2010), and type 2 diabetes (Aschner, 2010). Obesity may also be a risk factor for migraine, especially chronic migraine.

## Metabolic Syndrome, Insulin Resistance, and Migraine

Although studies of metabolic syndrome components (hypertension, hyperlipidemia, insulin resistance, adiposity) and migraine exist, only one study characterized metabolic syndrome patients with migraine. Guldiken et al. (2009) surveyed 210 patients who met Adult Treatment Panel 3 (ATP III) criteria for metabolic syndrome. They assessed headache symptoms lasting more than a year and meeting International Classification of Headache Disorders-II (ICHD-II) criteria for migraine. Patients with migraine and metabolic syndrome (*n* = 41, 19.5%) were significantly more likely to have diabetes, increased waist circumference, and elevated body-mass index than patients with metabolic syndrome without migraine. These findings indicate that insulin resistance may be associated with migraine pathogenesis. Several lines of evidence support this theory. Studies of migraine triggers indicate that 46.3% (Yadav et al., 2010) – 57.3% (Hauge et al., 2010) of migraineurs associate fasting with migraine onset. Additionally, two clinic-based population studies of ICHD-II–defined adult migraineurs found that insulin resistance is significantly altered in (1) young, non-obese, non-diabetic, normotensive migraine patients as opposed to healthy control subjects (Rainero et al., 2005), and (2) migraine patients compared with non-migraine headache patients (Cavestro et al., 2007). One group reported a link between insulin receptor gene polymorphism at locus 19p13.3/2 and migraine with aura (McCarthy et al., 2001; Kaunisto et al., 2005; Curtain, 2006; Netzer et al., 2008), although it is unclear if this polymorphism confers a loss or gain of function. While insulin receptors are found in many regions of the brain, the signals that these receptors propagate and the effect of resistance on their activity remains unclear. The clinical characteristics of migraine itself may lead to metabolic syndrome. Physical activity usually worsens migraine symptoms during acute attacks, which could lead patients to avoid exercise (ICHD-2 criteria for migraine). Affective disorders or medications used to treat migraine, such as antidepressants, commonly cause weight gain (Young and Rozen, 2005; Table 1). Even topiramate, which tends to decrease weight, may worsen or cause dyslipidemia (Franzoni et al., 2007).

**Table 1:** **Select migraine prophylactic medications and accompanying metabolic effects**.

Migraine type	Metabolic derangement	Reference
Migraine NOS	Increased insulin resistance	Rainero et al. (2005)
Migraine NOS	Increased systolic blood pressure	Buse et al. (2010)
Migraine NOS	Increased serum IL-6	Recober and Goadsby (2010)
Chronic migraine	Increased systolic blood pressure	Barbanti et al. (2010)/Bigal (2010)
Episodic migraine	Decreased serum leptin	Guldiken et al. (2008)
Menstrual migraine	Increased serum TNF-alpha	Mueller et al. (2001)
Chronic daily headache	Increased systolic blood pressure	Gipponi et al. (2010)

## Systemic Blood Pressure and Migraine

A few studies have reported the presence of blood pressure abnormalities (systolic or diastolic) in migraine. Hypertension is clearly linked to cerebrovascular disease (Netzer et al., 2008) and has been associated with self-reported headache (subtype unspecified; Cirillo et al., 1999). Severe headache is a common symptom of a hypertensive emergency. Blood pressure abnormalities have been associated with migraine in pregnancy (Adeney and Williams, 2006), migraine with aura, and chronic migraine (Granella et al., 1987). Migraine is a risk factor for pre-eclampsia and gestational hypertension (Allais et al., 2010). Studies of hypertension in non-pregnant adults with migraine are conflicting. A large demographic study positively associated migraine with systolic hypertension (Buse et al., 2010). However, other studies have associated systolic hypertension with chronic daily headache (Gipponi et al., 2010) and chronic migraine (Bigal et al., 2002; Barbanti et al., 2010), but not episodic migraine or tension-type headache in non-pregnant adult patients. Diastolic hypertension has been associated with increased frequency of episodic migraine in one study (Gudmundsson et al., 2006) but diastolic *hypotension* was non-significantly associated with migraine in another study (Seçil et al., 2010). Some have suggested that autonomic dysfunction is an important part of migraine; many migraineurs exhibit symptoms, such as diarrhea or sweating, of parasympathetic over-activity (Melek et al., 2007). Further study will be required to assess the role of blood pressure abnormalities in migraine.

## Migraine and Obesity

Obesity is a major public health problem (Friedman, 2009). Obesity has been associated with numerous pain syndromes, including chronic pain (Ray et al., 2010), fibromyalgia (Okifuji et al., 2010), low back pain (Heuch et al., 2010), and neck pain (Mäntyselkä et al., 2010). Migraine patients may have multiple metabolic abnormalities associated with obesity, including cerebrospinal fluid (CSF) neuropeptide Y elevation (Valenzuela et al., 2000), CSF tumor necrosis factor alpha (TNF) elevation (Rozen and Swidan, 2007), and systemic adiponectin depression (Peterlin et al., 2007). Given the recently characterized metabolic activity of adipose tissue (Bigal et al., 2007a), the interaction between obesity and migraine is particularly complex and has been the subject of multiple large and conflicting studies.

Population-based studies suggest that obesity is not associated with migraine prevalence (Bigal et al., 2006b) but may be a risk factor for the transformation of episodic migraine to chronic migraine (Scher et al., 2003; Bigal and Lipton, 2006). Bigal et al. studied 30,215 subjects, 3,791 of whom reported migraine symptoms. In age-, education-, and race-adjusted models, migraine prevalence was not significantly associated with elevated body-mass index (BMI). However, increasing weight was associated with increasing headache frequency, severity, and disability (Bigal et al., 2006a). Bigal et al. (2007b) further identified 18,968 migraine patients from a validated, mailed survey and compared them to patients with probable migraine and severe episodic tension-type headache. Bigal et al. (2007b) found that BMI and headache frequency and disability were positively correlated in the migraine patient population but not in other headache groups. Winter et al. (2009) confirmed these findings in a survey of 63,467 women age >45 years, wherein they found that women with a high BMI (morbid obesity) and current (as opposed to historical) migraine attacks were more likely to suffer from more frequent migraine (OR 3.11 for daily migraine vs. lower BMI groups) and migraines with phonophobia and photophobia but not with aura. Finally, Tietjen et al. (2007) studied 721 migraine patients recruited from eight study centers and found that patients with migraine, obesity, depression, and anxiety had greater migraine frequency and migraine-related disability.

Several studies failed to find any association between migraine and obesity. Keith et al. (2008) surveyed 11 independent datasets totaling 220,370 females with headache, reporting no association between diagnosed migraine and BMI. Molarius et al. (2008) found no association between obesity and self-reported migraine in a survey of 43,770 patients. Mattsson (2007) studied 684 females age 40–74 and did not find any association between obesity and migraine prevalence, frequency, severity, or disability. Tellez-Zenteno et al. (2010) surveyed 1,371 migraine patients and 612 age- and gender-matched controls. They found that migraine patients were more likely to be overweight but less likely to be obese or morbidly obese (Tellez-Zenteno et al., 2010). They additionally did not find any association between weight and headache severity or frequency (Tellez-Zenteno et al., 2010). Unfortunately, many patients with migraine are unaware of their diagnosis, often labeling frequent headaches as “sinus” or “stress” headaches (Eross et al., 2007).

A number of smaller studies found an association between BMI and migraine prevalence. Peterlin et al. (2010) proposed that differences in visceral as opposed to subcutaneous adipose tissue may help explain sex differences in migraine prevalence. Women after menopause and men both tend to have more abdominal obesity stored in visceral tissue, placing them at increased risk for hypertension, hyperlipidemia, and cardiovascular events. Subcutaneous fat, often in the gluteo-femoral region in women, appears to increase leptin and adiponectin levels, which may impair insulin sensitivity and modulation of inflammatory processes contributing to migraine risk. In a 7,601 patient sub-population of the National Health and Nutrition Examination Survey, Ford et al. (2008) reported that overweight and underweight patients were more likely to suffer from migraine. Horev et al. (2005) interviewed 27 morbidly obese women patients, reporting migraine symptoms in 13 patients (10 with aura) and tension-type headache in an additional 4 patients. In the adolescent population, Pinhas-Hamiel et al. (2008) surveyed 273 individuals and uncovered 39 children with headache and 15 with migraine. Increasing weight was associated with increased headache prevalence (Pinhas-Hamiel et al., 2008). Of note, migraine patients tend to significantly underestimate self-reported weight and BMI when compared with actual measurements (Katsnelson et al., 2009).

### The metabolic activity of excess adipose tissue likely contributes to a proinflammatory state, contributing to migraine pathogenesis

While the relationship between migraine and obesity is controversial, most large studies suggest that obesity is not associated with migraine prevalence, but may be associated with increased headache frequency.

Multiple neurotransmitters and hormones, including CGRP, substance P, neuropeptide Y (Valenzuela et al., 2000), adiponectin (Peterlin et al., 2007), leptin (Guldiken et al., 2008), orexin-A (Ray and Kumar, 2010), interleukin (IL)-1 (Yilmaz et al., 2010), IL-6, and TNF-α have been implicated in migraine pathogenesis (Rozen and Swidan, 2007; Yilmaz et al., 2010). Adipocytes and associated macrophages are known to be metabolically active (Bigal et al., 2007a), producing many mediators of migraine. Adiponectin and leptin, hormones involved in the regulation of appetite and metabolism, play a central role in adipose tissue development and subsequent aberrations in the metabolic milieu of obesity (Arita et al., 1999; Stofkova, 2009). Serum adiponectin levels have been inversely correlated with BMI (Arita et al., 1999) and adiponectin may mediate many of the inflammatory metabolites associated with migraine (Peterlin et al., 2007). Leptin is an adipose-derived hormone that helps regulate appetite and metabolism, produced by adipose tissue and increased in obese patients. Leptin acts on receptors in the hypothalamus combating the effect of feeding stimulants, such as neuropeptide Y, reducing appetite (Peterlin, 2009). Elevated serum leptin is also known to enhance both innate and adaptive immune system activity (Stofkova, 2009). In one study of episodic migraine subjects, Leptin was decreased compared to controls with similar BMI (Guldiken et al., 2008), but the duration of disease may be an overlooked factor. In patients with acute inflammation, levels are increased, but they decrease with chronic exposure to inflammation, suggesting that patients with a long history or frequent migraine would be at greatest risk of weight gain (Peterlin, 2009).

Vascular dilation and inflammation are thought to contribute to migraine pathogenesis and pain symptoms. Inflammatory and immune mediators, including TNF-α, IL-1, CGRP, orexin, and IL-6, are involved in migraine pathogenesis, and substance P, CGRP, and IL-6 are elevated during acute migraine attacks (Jang et al., 2010). TNF-α is a proinflammatory molecule produced by excess adipose tissue (Rönnemaa et al., 2000) and serum TNF-α levels are elevated in menstrual migraine (Mueller et al., 2001). The gain of function TNF-α-308 A and IL-1β + 3953 T allele gene polymorphisms are elevated in patients with migraine without aura (Yilmaz et al., 2010), although the implications are unclear. CGRP is a neurotransmitter that mediates nociceptive transmission from the trigeminal nerve complex to the thalamus (Summ et al., 2010). Elevated CGRP levels have been detected in obese patients, conferring further risk of migraine in susceptible patients (Recober and Goadsby, 2010). Similarly, IL-6 is elevated in migraine patients regardless of current treatment protocol and is thought to lower the pain thresholds. Orexin-A and B receptors have been studied in migraine. Orexin-A is thought to depress CGRP release (Holland et al., 2005), and orexin activation is thought to confer resistance to dietary related features of metabolic syndrome (Funato et al., 2009). Migraine and obesity independently give rise to an altered inflammatory and immune marker milieu, and further research will be required to elucidate how these two diseases interact.

### It is unclear if obesity causes migraine or if migraine causes obesity

Obesity, like other comorbid states in migraine, is a result of genetic and environmental susceptibility to disease. It is unclear if migraine, or its treatment, is a cause of obesity, or if obesity causes increased migraine frequency. Exercise increases endorphin levels and pain tolerance and in rats decreases opioid usage (Hosseini et al., 2009) and increases insulin sensitivity (Su et al., 2005). Physical activity tends to exacerbate symptoms of migraine, so it is likely that patients with frequent migraine would avoid strenuous activity. In most studies, obesity does not predispose to increased migraine prevalence, but it could be a risk factor for more frequent or severe attacks.

## Migraine and Prothrombotic State

Metabolic syndrome is associated with an acquired prothrombotic state. Thrombosis is a leading cause of multiple cardiovascular and cerebrovascular disease processes. Migraine, particularly migraine with aura, is associated with an increased risk of cardiovascular and cerebrovascular disease in epidemiological studies (Kurth et al., 2006; Allais et al., 2008); however, objective ultrasound-based measures of atherosclerosis do not support a greater atherosclerotic burden in migraineurs when compared with headache-free controls (Moschiano et al., 2007). Migraine without aura is not associated with an increased burden of vascular disease (Kurth et al., 2006). Certain migraine syndromes, particularly in migraine with aura, are associated with computed tomography and magnetic resonance imagining findings, including infarct-like white matter changes (Moschiano et al., 2007). These infarcts may accumulate over time as seen on follow-up studies (Kruit et al., 2010). The cause of these findings are unclear, but seem to correlate with attack frequency. One possibility is that spreading oligemia during migraine aura may lead to subclinical ischemia. There is no evidence to support anticoagulation in migraine and it is unclear whether an acquired prothrombotic state would increase the prevalence of migraine.

Gain of function platelet glycoprotein polymorphisms have been associated with a minor increase in risk of migraine; however, these polymorphisms have been more strongly associated with multiple neurologic phenomena and a putative mechanism for the association with migraine requires further study (Herak et al., 2009). In the pediatric migraine population, factor V Leiden, factor II G20210A, methylenetetrahydrofolate reductase (MTHFR) C677T, and A1298C mutation frequency are not significantly associated with migraine; however elevated homocysteine and depressed folate levels may be (Bottini et al., 2006). Except in cases with antiphospholipid antibody syndrome (Asherson et al., 2007) and patient foramen ovale (PFO; Wilmshurst et al., 2005), anticoagulation or antithrombotic agents do not appear to prevent migraine.

Adult and pediatric patients with migraine with aura are significantly more likely to have PFO than those in the general population. PFO-associated microembolization of cortical vasculature may contribute to the initiation of cortical spreading depression in migraine with aura (Rigatelli et al., 2010). An observational, prospective study reports that 89% of migraine patients with PFO improved following closure, with 46% reporting cure at 12 months (Trabattoni et al., 2011). However, a randomized, double-blind, sham-controlled trial did not find a significant association from PFO closure and improved migraine symptoms (Dowson et al., 2008).

## Clinical Implications

Migraine treatment may be acute or preventive, and patients with severe, frequent attacks may require both. Because patients with chronic migraine have frequent or even daily headaches, preventive treatments are key, and preventive treatments, such as weight loss and behavior modification, are critical to avoiding the development of metabolic syndrome and managing and avoiding the cardiovascular implications of the disease (Bond et al., 2010). However, significant controversy exists regarding the relationship between metabolic syndrome and migraine and the effect that treating one has on abating the other. Further, the subcomponents of metabolic syndrome, including insulin resistance, elevated blood pressure, obesity, proinflammatory state, and prothrombotic state, have an unclear association with migraine prevalence, frequency, and characteristics (Table 2). It is unknown if treating metabolic syndrome risk factors results in a decrease in migraine attack rates or duration, or susceptibility to migraine development. Finally, the prevalence of metabolic syndrome in the migraine population has not been assessed.

**Table 2:** **Select metabolic derangements found in migraine by migraine type**.

Drug class	Medication	Metabolic effect	Reference
Antiepileptic	Topiramate	Weight loss, dyslipidemia	Franzoni et al. (2007)
Tricyclic antidepressant	Amitriptyline	Reduction in serum orexin-A and orexin-B levels. Increase in serum neuropeptide Y. Weight gain. Increased serum leptin, C-peptide, and insulin insulin	Caproni et al. (2011), Berilgen et al. (2005)
Beta-adrenergic receptor blocker	Propranolol	Weight gain, worsening of glycemic and lipidic control (non-vasodilating beta-blockers as a drug class)	Sharma et al. (2001), Fonseca (2010)
SSRI/SNRI	Venlafaxine	Weight gain, hypertension at high dose	Harrison et al. (2004)
Antiepileptic	Valproic acid and derivatives	Increased BMI, serum insulin, leptin, neuropeptide Y	Tokgoz et al. (2012), Pylvänen et al. (2002)

*NOS = Not otherwise specified*.

### Migraine diets, food avoidance, and supplements may be useful in migraine, but these treatments are not specific to migraineurs with metabolic syndrome

While food related headache triggers are well documented (Alpay et al., 2010) the use of so-called “migraine diets,” designed to minimize migraine frequency and food sensitization, has been controversial (Damen et al., 2006). One small trial of 30 patients with migraine without aura does support food avoidance based on detection of circulating serum immunoglobulins against specific foods; however, larger studies are required to determine the efficacy of this technique (Alpay et al., 2010). Four non-prescription supplements, including magnesium, *Petasites hybridus*, coenzyme Q10, and riboflavin, have been found to be effective migraine preventive agents based on double-blind placebo-controlled trials (Sun-Edelstein and Mauskop, 2009). However, supplementation is not intended exclusively for the obese population.

### Sleep disturbance is associated with increased headache severity but not specifically migraine in patients with metabolic syndrome

Obesity can cause sleep abnormalities (Kopelman, 2000), which may worsen headaches and general health (Rains, 2008). In particular, obstructive sleep apnea and sleep disturbance are associated with chronic migraine and chronic daily headache (Rains and Poceta, 2006). Obstructive sleep apnea is also associated with metabolic syndrome (Bhushan et al., 2010). Weight loss is frequently curative in obese patients with obstructive sleep apnea. In patients who do not lose weight, continuous positive airway pressure therapy appears to reduce apnea-associated headache frequency (Rains and Poceta, 2010). Further, good sleep hygiene may reduce migraine frequency (Gilman et al., 2007).

### Medication choice in migraine prevention may have implications for weight gain and concomitant metabolic syndrome management

Numerous medications used in the treatment of migraine, including tricyclic antidepressants (i.e., amitriptyline; Taylor, 2008), beta-adrenergic blockers (i.e., propranolol; Taylor, 2008), antiepileptic drugs (e.g., divalproex sodium and gabapentin; Taylor, 2008), and selective serotonin and norepinephrine reuptake inhibitors, cause weight gain (Young, 2008). Although their headaches improve with preventive migraine treatment (Bigal et al., 2006a), fear of gaining weight is the most common reason patients stop taking their migraine preventive medication (Kowacs et al., 2009). Weight gain from migraine therapy is associated with a significant increase in the cardiovascular risk factors associated with metabolic syndrome (Bigal et al., 2009).

Topiramate is a popular preventive migraine treatment (Kowacs et al., 2009). Topiramate monotherapy is at least as effective as amitriptyline monotherapy in preventing headaches and improving functionality (Dodick et al., 2009), and it is hypothesized to cause centrally mediated weight loss (Schütt et al., 2010). Topiramate has been suggested as a single-agent short-term adjuvant to exercise and caloric restriction regimens for weight loss in the non-headache population (Bays, 2010). This indication is off-label and requires further study; particularly because a high BMI at baseline is not predictive of which migraine patients will lose weight on topiramate (Alberici et al., 2009). Topiramate monotherapy in migraine has been associated with significant elevations of serum uric acid, increasing risk of nephrolithiasis, and non-significant serum triglyceride elevation (Koçer et al., 2008).

OnabotulinumtoxinA (BTX) is also effective in migraine prophylaxis, particularly chronic migraine (Aurora et al., 2010). BTX is potentially very useful in patients whose compliance is poor or in whom other medications are contraindicated. BTX does not cause weight gain and systemic adverse events are rare (Gobel, 2004).

Migraine and concomitant depression is a well described phenomenon, and in many cases depression is secondary to the migraine patient’s decreased quality of life. However, recent work appears to suggest that depression itself may confer a significant risk of migraine and that serotonin may mediate both processes (Marino et al., 2010). Despite the potential interrelated pathophysiology of migraine and depression, and the utility of tricyclic antidepressants in migraine prophylaxis, the efficacy of the SSRI class of medications in migraine prevention has been questioned (Moja et al., 2005). One case report suggested that SSRIs may worsen acute migraine attacks (Bickel et al., 2005). SSRIs and SNRIs are known to cause weight gain (Young, 2008). However, these classes of medications may provide adequate migraine prophylaxis for patients who cannot tolerate tricyclic antidepressants and have concomitant (or primary) depression. Patients with depression have higher rates of metabolic syndrome than non-depressed control subjects (East et al., 2010). It is not known whether depression or medication-related weight gain confers a greater risk of developing metabolic syndrome; however, treating depression is known to improve quality of life (Culpepper, 2010).

### Physical activity is effective in reducing risk of metabolic syndrome and inactivity may be associated with migraine

Several large population studies suggest that reduced physical activity is associated with migraine. Milde-Busch et al. (2010) surveyed 1,260 10th and 11th grade adolescents for ICHD-II–criteria migraine and tension-type headache and found a 3.4× risk of self-reported migraine symptoms in subjects with physical inactivity. In the HUNT study, Robberstad et al. (2010) reported that, among a subset of 5,847 adolescents assessed for recurrent headache symptoms (migraine or tension-type), physical activity, smoking status, and weight, overweight subjects were 1.4× more likely to report recurrent headache and those with low physical activity were 1.2× more likely to report recurrent headache. In a broader cross sectional survey of 46,648 individuals, also performed by the HUNT study group, low physical activity (sweating or exercising during leisure time to the point of being out of breath less than 1 h per week) was associated with a higher incidence of self-reported migraine and non-migraine headache (Varkey et al., 2008). A single patient case report suggests that intense running may abort an evolving migraine, though this has yet to be reproduced (Strelniker, 2009). Similarly, a high level of physical activity may confer a reduced risk of metabolic syndrome (Dai et al., 2010).

Several studies have evaluated aerobic exercise programs in migraine patients (Köseoglu et al., 2003; Dittrich et al., 2008; Varkey et al., 2009). These programs have focused on increasing oxygen uptake and have been well tolerated except for rare post-exercise migraines (Varkey et al., 2009). Aerobic programs have demonstrated improved depression (Dittrich et al., 2008) and pain severity (Lockett and Campbell, 1992) scores. It is hypothesized that aerobic exercise programs are associated with basal beta endorphin levels, potentially modifying pain sensation threshold (Köseoglu et al., 2003). For patients with metabolic syndrome, exercise is more complicated. Studies reviewing exercise protocols for obese patients and in rats indicate that a mix of resistance and endurance training may be indicated to improve cardiovascular disease risk endpoints (Hansen et al., 2010; Touati et al., 2010).

## Conclusion

Metabolic syndrome and migraine are highly complex biochemical processes. It is likely that the two interact; however, it is unclear how they do. Further research will be required to elucidate the best clinical practices in patients who present with both diseases. Weight loss, good sleep hygiene, increased physical activity, and management of life stressors are the cornerstone of preventive healthcare in the adult population. These should be encouraged in all patients, although the exact benefits in patients with migraine and metabolic syndrome are unclear. Preventive treatments, including nutraceuticals, pharmaceuticals, and avoidance of triggers, are commonly accepted migraine treatments. The choice of pharmaceutical may have implications for the management of metabolic syndrome; however, much has yet to be learned about the long-term use of all preventive medications. Consultation with the patient regarding pharmacologic side effects is necessary.

## Conflict of Interest Statement

Dr. Amit Sachdev has nothing to disclose. Dr. Michael J. Marmura has received support for clinical research from Merck and received compensation for advisory consultations from NeurogesX and Iroko.

## References

[B1] Adeney K. L. Williams M. (2006). Migraine headaches and preeclampsia: an epidemiologic review. Headache 46, 794–803 10.1111/j.1526-4610.2006.00432.x 16643583

[B2] Aderjan D. Stankewitz A. May A. (2010). Neuronal mechanisms during repetitive trigemino-nociceptive stimulation in migraine patients. Pain 151, 97–103 10.1016/j.pain.2010.06.024 20638178

[B3] Alberici A. Borroni B. Manelli F. Griffini S. Zavarise P. Padovani A. (2009). Topiramate weight loss in migraine patients. J. Neurol. Sci. 278, 64–65 10.1016/j.jns.2008.11.014 19084843

[B4] Alberti K. G. Zimmet P. Shaw J. (2006). Metabolic syndrome – a new world-wide definition. A consensus statement from the International Diabetes Federation. Diabet. Med. 23, 469–480 10.1111/j.1464-5491.2006.01994_2.x 16681555

[B5] Allais G. Gabellari I. Borgogno P. De Lorenzo C. Benedetto C. (2010). The risks of women with migraine during pregnancy. Neurol. Sci. 31(Suppl. 1), S59–S61 10.1007/s10072-010-0323-4 20464585

[B6] Allais G. Gabellari I. Mana O. Schiapparelli P. Terzi M. G. Benedetto C. (2008). Migraine and stroke: the role of oral contraceptives. Neurol. Sci. 29(Suppl. 1), S12–S14 10.1007/s10072-008-0921-6 18545887

[B7] Alpay K. Erta M. Orhan E. K. Ustay D. K. Lieners C. Baykan B. (2010). Diet restriction in migraine, based on IgG against foods: a clinical double-blind, randomised, cross-over trial. Cephalalgia 30, 829–837 10.1177/0333102410361404 20647174 PMC2899772

[B8] Anscombe R. Krebs J. Weatherall M. Harding S. A. (2006). Redefinition of the metabolic syndrome – useful or creating illness? N. Z. Med. J. 119, U2372 17195862

[B9] Arita Y. Kihara S. Ouchi N. Takahashi M. Maeda K. Miyagawa J. (1999). Paradoxical decrease of an adipose-specific protein, adiponectin, in obesity. Biochem. Biophys. Res. Commun. 257, 79–83 10.1006/bbrc.1999.0255 10092513

[B10] Aschner P. (2010). Metabolic syndrome as a risk factor for diabetes. Expert Rev. Cardiovasc. Ther. 8, 407–412 10.1586/erc.10.13 20222818

[B11] Asherson R. A. Giampaulo D. Singh S. Sulman L. (2007). Dramatic response of severe headaches to anticoagulation in a patient with antiphospholipid syndrome. J. Clin. Rheumatol. 13, 173–174 10.1097/RHU.0b013e3180690af6 17551389

[B12] Aurora S. Kori S. Barrodale P. Nelsen A. McDonald S. (2007). Gastric stasis occurs in spontaneous, visually induced, and interictal migraine. Headache 47, 1443–1446 10.1111/j.1526-4610.2007.00853.x 17868348

[B13] Aurora S. K. Dodick D. Turkel C. C. DeGryse R. E. Silberstein S. D. Lipton R. B. (2010). OnabotulinumtoxinA for treatment of chronic migraine: results from the double-blind, randomized, placebo-controlled phase of the PREEMPT 1 trial. Cephalalgia 30, 793–803 10.1177/0333102410364676 20647170

[B14] Ayata C. (2010). Cortical spreading depression triggers migraine attack: pro. Headache 50, 725–730 10.1111/j.1526-4610.2010.01647.x 20456160

[B15] Ba’albaki H. Rapoport A. (2008). Mast cells activate the renin angiotensin system and contribute to migraine: a hypothesis. Headache 1499–1505 10.1111/j.1526-4610.2008.00852.x 19076648

[B16] Barbanti P. Aurilia C. Egeo G. Fofi L. (2010). Hypertension as a risk factor for migraine chronification. Neurol. Sci. 31(Suppl. 1), S41–S43 10.1007/s10072-009-0167-y 20464581

[B17] Bays H. (2010). Phentermine, topiramate and their combination for the treatment of adiposopathy (‘sick fat’) and metabolic disease. Expert Rev. Cardiovasc. Ther. 8, 1777–1801 10.1586/erc.10.125 20707765

[B18] Berilgen M. S. Bulut S. Gonen M. Tekatas A. Dag E. Mungen B. (2005). Comparison of the effects of amitriptyline and flunarizine on weight gain and serum leptin, C peptide and insulin levels when used as migraine preventive treatment. Cephalalgia 25, 1048–1053 10.1111/j.1468-2982.2005.00956.x 16232156

[B19] Bhushan B. Misra A. Guleria R. (2010). Obstructive sleep apnea is independently associated with the metabolic syndrome in obese Asian Indians in northern India. Metab. Syndr. Relat. Disord. 8, 431–435 10.1089/met.2009.0125 20715932

[B20] Bickel A. Kornhuber J. Maihöfner C. Ropohl A. (2005). Exacerbation of migraine attacks during treatment with the selective serotonin reuptake inhibitor sertraline. A case report. Pharmacopsychiatry 38, 327–328 10.1055/s-2005-916190 16342007

[B21] Bigal M. E. (2010). Migraine and cardiovascular disease. A call for action. Headache 50, 882–883 10.1111/j.1526-4610.2010.01717.x 20546322

[B22] Bigal M. E. Gironda M. Tepper S. J. Feleppa M. Rapoport A. M. Sheftell F. D. (2006a). Headache prevention outcome and body mass index. Cephalalgia 26, 445–450 10.1111/j.1468-2982.2006.01086.x 16556246

[B23] Bigal M. E. Liberman J. Lipton R. B. (2006b). Obesity and migraine: a population study. Neurology 66, 545–550 10.1212/01.wnl.0000197218.05284.82 16354886

[B24] Bigal M. E. Lipton R. (2006). Obesity is a risk factor for transformed migraine but not chronic tension-type headache. Neurology 67, 252–257 10.1212/01.wnl.0000225052.35019.f9 16864817

[B25] Bigal M. E. Lipton R. Biondi D. M. Xiang J. Hulihan J. (2009). Weight change and clinical markers of cardiovascular disease risk during preventive treatment of migraine. Cephalalgia 29, 1188–1196 10.1111/j.1468-2982.2009.01853.x 19558539

[B26] Bigal M. E. Lipton R. Holland P. R. Goadsby P. J. (2007a). Obesity, migraine, and chronic migraine: possible mechanisms of interaction. Neurology 68, 1851–1861 10.1212/01.wnl.0000262045.11646.b1 17515549

[B27] Bigal M. E. Tsang A. Loder E. Serrano D. Reed M. L. Lipton R. B. (2007b). Body mass index and episodic headaches: a population-based study. Arch. Intern. Med. 167, 1964–1970 10.1001/archinte.167.18.1964 17923596

[B28] Bigal M. E. Sheftell F. Rapoport A. M. Tepper S. J. Lipton R. B. (2002). Chronic daily headache: identification of factors associated with induction and transformation. Headache 42, 575–581 10.1046/j.1526-4610.2002.02143.x 12482208

[B29] Blau J. N. (1992). Migraine: theories of pathogenesis. Lancet 339, 1202–1207 10.1016/0140-6736(92)91140-4 1349944

[B30] Bond D. S. Roth J. Nash J. M. Wing R. R. (2010). Migraine and obesity: epidemiology, possible mechanisms and the potential role of weight loss treatment. Obes. Rev. 12, e362–e371 10.1111/j.1467-789X.2010.00791.x 20673279 PMC2974024

[B31] Bottini F. Celle M. Calevo M. G. Amato S. Minniti G. Montaldi L. (2006). Metabolic and genetic risk factors for migraine in children. Cephalalgia 26, 731–737 10.1111/j.1468-2982.2006.01107.x 16686913

[B32] Buse D. C. Manack A. Serrano D. Turkel C. Lipton R. B. (2010). Sociodemographic and comorbidity profiles of chronic migraine and episodic migraine sufferers. J. Neurol. Neurosurg. Psychiatr. 81, 428–432 10.1136/jnnp.2009.192492 20164501

[B33] Buzzi M. G. Tassorelli C. (2010). Experimental models of migraine. Handb. Clin. Neurol. 97, 109–123 10.1016/S0072-9752(10)97008-5 20816414

[B34] Caproni S. Corbelli I. Pini L. A. Cupini M. L. Calabresi P. Sarchielli P. (2011). Migraine preventive drug-induced weight gain may be mediated by effects on hypothalamic peptides: the results of a pilot study. Cephalalgia 31, 543–549 10.1177/0333102410392605 21216871

[B35] Cavestro C. Rosatello A. Micca G. Ravotto M. Marino M. P. Asteggiano G. (2007). Insulin metabolism is altered in migraineurs: a new pathogenic mechanism for migraine? Headache 47, 1436–1442 10.1111/j.1526-4610.2007.00719.x 18052953

[B36] Cirillo M. Stellato D. Lombardi C. De Santo N. G. Covelli V. (1999). Headache and cardiovascular risk factors: positive association with hypertension. Headache 39, 409–416 10.1046/j.1526-4610.1999.3906409.x 11279918

[B37] Culpepper L. (2010). Why do you need to move beyond first-line therapy for major depression? J. Clin. Psychiatry 71(Suppl. 1), 4–9 10.4088/JCP.8139tx4cc 20977873

[B38] Curtain R. (2006). No mutations detected in the INSR gene in a chromosome 19p13 linked migraine pedigree. Eur. J. Med. Genet. 49, 57–62 10.1016/j.ejmg.2005.01.015 16473310

[B39] Dai D. F. Hwang J. Chen C. L. Chiang F. T. Lin J. L. Hsu K. L. (2010). Effect of physical activity on the prevalence of metabolic syndrome and left ventricular hypertrophy in apparently healthy adults. J. Formos. Med. Assoc. 109, 716–724 10.1016/S0929-6646(10)60116-7 20970068

[B40] Damen L. Bruijn J. Koes B. W. Berger M. Y. Passchier J. Verhagen A. P. (2006). Prophylactic treatment of migraine in children. Part 1. A systematic review of non-pharmacological trials. Cephalalgia 26, 373–383 10.1111/j.1468-2982.2005.01047.x 16556238

[B41] Dittrich S. M. Gunther V. Franz G. Burtscher M. Holzner B. Kopp M. (2008). Aerobic exercise with relaxation: influence on pain and psychological well-being in female migraine patients. Clin. J. Sport Med. 18, 363–365 10.1097/JSM.0b013e31817efac9 18614890

[B42] Dodick D. W. Freitag F. Banks J. Saper J. Xiang J. Rupnow M. (2009). Topiramate versus amitriptyline in migraine prevention: a 26-week, multicenter, randomized, double-blind, double-dummy, parallel-group noninferiority trial in adult migraineurs. Clin. Ther. 31, 542–559 10.1016/j.clinthera.2009.03.020 19393844

[B43] Dowson A. Mullen M. Peatfield R. Muir K. Khan A. A. Wells C. (2008). Migraine intervention with STARFlex technology (MIST) trial: a prospective, multicenter, double-blind, sham-controlled trial to evaluate the effectiveness of patent foramen ovale closure with STARFlex septal repair implant to resolve refractory migraine headache. Circulation 117, 1397–1404 10.1161/CIRCULATIONAHA.107.727271 18316488

[B44] East C. Willis B. Barlow C. E. Grannemann B. D. FitzGerald S. J. DeFina L. F. (2010). Depressive symptoms and metabolic syndrome in preventive healthcare: the Cooper Center longitudinal study. Metab. Syndr. Relat. Disord. 8, 451–457 10.1089/met.2010.0017 20854094

[B45] Eross E. Dodrick D. Eross M. (2007). The sinus, allergy and migraine study (SAMS). Headache 47, 213–224 10.1111/j.1526-4610.2006.00688.x 17300361

[B46] Fonseca V. A. (2010). Effects of beta-blockers on glucose and lipid metabolism. Curr. Med. Res. Opin. 26, 615–629 10.1185/03007990903533681 20067434

[B47] Ford E. S. Li C. Pearson W. S. Zhao G. Strine T. W. Mokdad A. H. (2008). Body mass index and headaches: findings from a national sample of US adults. Cephalalgia 28, 1270–1276 10.1111/j.1468-2982.2008.01671.x 18727641

[B48] Franzoni E. Verrotti A. Sarajlija J. Garone C. Matricardi S. Salerno G. G. (2007). Topiramate: effects on serum lipids and lipoproteins levels in children. Eur. J. Neurol. 14, 1334–1337 10.1111/j.1468-1331.2007.01973.x 17916078

[B49] Friedman J. M. (2009). Obesity: causes and control of excess body fat. Nature 459, 340–342 10.1038/459340a 19458707

[B50] Funato H. Tsai A. Willie J. T. Kisanuki Y. Williams S. C. Sakurai T. (2009). Enhanced orexin receptor-2 signaling prevents diet-induced obesity and improves leptin sensitivity. Cell Metab. 9, 64–76 10.1016/j.cmet.2008.10.010 19117547 PMC2630400

[B51] Gilman D. K. Palermo T. Kabbouche M. A. Hershey A. D. Powers S. W. (2007). Primary headache and sleep disturbances in adolescents. Headache 47, 1189–1194 10.1111/j.1526-4610.2007.00885.x 17883524

[B52] Gipponi S. Venturelli E. Rao R. Liberini P. Padovani A. (2010). Hypertension is a factor associated with chronic daily headache. Neurol. Sci. 31(Suppl. 1), S171–S173 10.1007/s10072-010-0322-5 20464615

[B53] Gobel H. (2004). Botulinum toxin in migraine prophylaxis. J. Neurol. 251(Suppl. 1), I8–I11 10.1007/s00415-004-1103-y 14991336

[B54] Granella F. Farina S. Malferrari G. Manzoni G. C. (1987). Drug abuse in chronic headache: a clinico-epidemiologic study. Cephalalgia 7, 15–19 10.1046/j.1468-2982.1987.0701015.x 3581158

[B55] Gudmundsson L. S. Thorgeirsson G. Sigfusson N. Sigvaldason H. Johannsson M. (2006). Migraine patients have lower systolic but higher diastolic blood pressure compared with controls in a population-based study of 21,537 subjects. The Reykjavik study. Cephalalgia 26, 436–444 10.1111/j.1468-2982.2005.01057.x 16556245

[B56] Guldiken B. Guldiken S. Demir M. Turgut N. Tugrul A. (2008). Low leptin levels in migraine: a case control. Headache 48, 1103–1107 10.1111/j.1526-4610.2008.01152.x 18547265

[B57] Guldiken B. Guldiken S. Taskiran B. Koc G. Turgut N. Kabayel L. (2009). Migraine in metabolic syndrome. Neurologist 15, 55–58 10.1097/NRL.0b013e31817781b6 19276782

[B58] Gupta A. K. Dahlof B. Sever P. S. Poulter N. R. Anglo-Scandinavian Cardiac Outcomes Trial-Blood Pressure Lowering Arm Investigators (2010). Metabolic syndrome, independent of its components, is a risk factor for stroke and death but not for coronary heart disease among hypertensive patients in the ASCOT-BPLA. Diabetes Care 33, 1647–1651 10.2337/dc09-2208 20413525 PMC2890375

[B59] Hansen D. Dendale P. van Loon L. J. Meeusen R. (2010). The impact of training modalities on the clinical benefits of exercise intervention in patients with cardiovascular disease risk or type 2 diabetes mellitus. Sports Med. 40, 921–940 10.2165/11535930-000000000-00000 20942509

[B60] Harrison C. L. Ferrier N. Young A. H. (2004). Tolerability of high-dose venlafaxine in depressed patients. J. Psychopharmacol. (Oxford) 18, 200–204 10.1177/0269881104040225 15260908

[B61] Hauge A. W. Kirchmann M. Olesen J. (2010). Characterization of consistent triggers of migraine with aura. Cephalalgia 31, 416–438 10.1177/0333102410382795 20847084

[B62] Herak D. C. Antolic M. Krleza J. L. Pavic M. Dodig S. Duranovic V. (2009). Inherited prothrombotic risk factors in children with stroke, transient ischemic attack, or migraine. Pediatrics 123, e653–e660 10.1542/peds.2008-1439 19336355

[B63] Heuch I. Hagen K. Heuch I. Nygaard Ø. Zwart J. A. (2010). The impact of body mass index on the prevalence of low back pain: the HUNT study. Spine 35, 764–768 20228714 10.1097/BRS.0b013e3181ba1531

[B64] Holland P. R. Akerman S. Goadsby P. J. (2005). Orexin 1 receptor activation attenuates neurogenic dural vasodilation in an animal model of trigeminovascular nociception. J. Pharmacol. Exp. Ther. 5, 1380–1385 10.1124/jpet.105.090951 16160082

[B65] Horev A. Wirguin I. Lantsberg L. Ifergane G. A. (2005). A high incidence of migraine with aura among morbidly obese women. Headache 45, 936–938 10.1111/j.1526-4610.2005.05162.x 15985113

[B66] Hosseini M. Alaei H. Naderi A. Sharifi M. R. Zahed R. (2009). Treadmill exercise reduces self-administration of morphine in male rats. Pathophysiology 16, 3–7 10.1016/j.pathophys.2008.11.001 19131225

[B67] Jang M. U. Park J. Kho H. S. Chung S. C. Chung J. W. (2010). Plasma and saliva levels of nerve growth factor and neuropeptides in chronic migraine patients. Oral. Dis. 17, 187–193 10.1111/j.1601-0825.2010.01717.x 20659258

[B68] Kahn R. Buse J. Ferrannini E. Stern M. (2005). The metabolic syndrome: time for a critical appraisal: joint statement from the American Diabetes Association and the European Association for the Study of Diabetes. Diabetes Care 28, 2289–2304 10.2337/diacare.28.9.2289 16123508

[B69] Katsnelson M. J. Peterlin B. Rosso A. L. Alexander G. M. Erwin K. L. (2009). Self-reported vs measured body mass indices in migraineurs. Headache 49, 663–668 10.1111/j.1526-4610.2009.01400.x 19472442 PMC3972494

[B70] Kaunisto M. A. Tikka P. Kallela M. Leal S. M. Papp J. C. Korhonen A. (2005). Chromosome 19p13 loci in Finnish migraine with aura families. Am. J. Med. Genet. B Neuropsychiatr. Genet. 132B, 85–89 10.1002/ajmg.b.30082 15449251 PMC6217809

[B71] Keith S. W. Wang C. Fontaine K. R. Cowan C. D. Allison D. B. (2008). BMI and headache among women: results from 11 epidemiologic datasets. Obesity 16, 377–383 10.1038/oby.2007.32 18239647 PMC3208164

[B72] Koçer A. Dikici S. Atakay S. Okuyucu S. (2008). Serum uric acid and lipid levels while taking topiramate for migraine. Headache 48, 1056–1060 10.1111/j.1526-4610.2007.01008.x 18179568

[B73] Kopelman P. G. (2000). Obesity as a medical problem. Nature 404, 635–643 10766250 10.1038/35007508

[B74] Köseoglu E. Akboyraz A. Soyuer A. Ersoy A. O. (2003). Aerobic exercise and plasma beta endorphin levels in patients with migrainous headache without aura. Cephalalgia 23, 972–976 10.1046/j.1468-2982.2003.00624.x 14984230

[B75] Kowacs P. A. Piovesan E. Tepper S. J. (2009). Rejection and acceptance of possible side effects of migraine prophylactic drugs. Headache 49, 1022–1027 10.1111/j.1526-4610.2009.01431.x 19438733

[B76] Kruit M. C. Van Buchem M. Launer L. J. Terwindt G. M. Ferrari M. D. (2010). Migraine is associated with an increased risk of deep white matter lesions, subclinical posterior circulation infarcts and brain iron accumulation: the population-based MRI CAMERA study. Cephalalgia 30, 129–136 19515125 10.1111/j.1468-2982.2009.01904.x PMC3241741

[B77] Kurth T. Gaziano J. Cook N. R. Logroscino G. Diener H. C. Buring J. E. (2006). Migraine and risk of cardiovascular disease in women. JAMA 296, 283–291 10.1001/jama.296.6.653-b 16849661

[B78] Lockett D. M. Campbell J. F. (1992). The effects of aerobic exercise on migraine. Headache 32, 50–54 10.1111/j.1526-4610.1992.hed3201050.x 1555933

[B79] Mäntyselkä P. Kautianinen H. Vanhala M. (2010). Prevalence of neck pain in subjects with metabolic syndrome – a cross-sectional population-based study. BMC Musculoskelet. Disord. 11, 171 10.1186/1471-2474-11-171 20670458 PMC2918543

[B80] Marino E. Fanny B. Lorenzi C. Pirovano A. Franchini L. Colombo C. (2010). Genetic bases of comorbidity between mood disorders and migraine: possible role of serotonin transporter gene. Neurol. Sci. 31, 387–391 10.1007/s10072-009-0183-y 19936882

[B81] Marmura M. J. (2009). Systemic abnormalities in migraine: what comes first. Neurologist 15, 53–54 10.1097/NRL.0b013e31817781e0 19276781

[B82] Mattsson P. (2007). Migraine headache and obesity in women aged 40-74 years: a population-based study. Cephalalgia 27, 877–880 10.1111/j.1468-2982.2007.01360.x 17635528

[B83] McCarthy L. C. Hosford D. Riley J. H. Bird M. I. White N. J. Hewett D. R. (2001). Single-nucleotide polymorphism alleles in the insulin receptor gene are associated with typical migraine. Genomics 78, 135–149 10.1006/geno.2001.6647 11735220

[B84] Melek I. M. Seyfeli E. Duru M. Duman T. Akgul F. Yalcin F. (2007). Autonomic dysfunction and cardiac repolarization abnormalities in patients with migraine attacks. Med. Sci. Monit. 13, RA47–RA49 17325646

[B85] Milde-Busch A. Blaschek A. Borggräfe I. Heinen F. Straube A. von Kries R. (2010). Associations of diet and lifestyle with headache in high-school students: results from a cross-sectional study. Headache 50, 1104–1114 10.1111/j.1526-4610.2009.01605.x 20533961

[B86] Milner P. M. (1958). Note on a possible correspondence between the scotomas of migraine and spreading depression of Leão. Electroencephalogr. Clin. Neurophysiol. 10, 705 10.1016/0013-4694(58)90073-7 13597818

[B87] Moja P. L. Cusi C. Sterzi R. R. Canepari C. (2005). Selective serotonin re-uptake inhibitors (SSRIs) for preventing migraine and tension-type headaches. Cochrane Database Syst. Rev. 20, CD002919 16034880 10.1002/14651858.CD002919.pub2

[B88] Molarius A. Tegelberg A. Ohrvik J. (2008). Socio-economic factors, lifestyle, and headache disorders – a population-based study in Sweden. Headache 48, 1426–1437 10.1111/j.1526-4610.2008.01178.x 18624712

[B89] Moschiano F. D’Amico A. D. Di Stefano M. Rocca N. Bussone G. (2007). The role of the clinician in interpreting conventional neuroimaging findings in migraine patients. Neurol. Sci. 28(Suppl.), S114–S117 10.1007/s10072-007-0799-8 17508156

[B90] Mottillo S. Filion K. Genest J. Joseph L. Pilote L. Poirier P. (2010). The metabolic syndrome and cardiovascular risk a systematic review and meta-analysis. J. Am. Coll. Cardiol. 56, 1113–1132 10.1016/j.jacc.2010.05.034 20863953

[B91] Mueller L. Gupta A. Stein T. P. (2001). Deficiency of tumor necrosis factor alpha in a subclass of menstrual migraineurs. Headache 41, 129–137 10.1046/j.1526-4610.2001.111006129.x 11251696

[B92] Netzer C. Freudenberg J. Heinze A. Heinze-Kuhn K. Goebel I. McCarthy L. (2008). Replication study of the insulin receptor gene in migraine with aura. Genomics 91, 503–507 10.1016/j.ygeno.2008.03.006 18455362

[B93] Okifuji A. Donaldson G. Barck L. Fine P. G. (2010). Relationship between fibromyalgia and obesity in pain, function, mood, and sleep. J. Pain 11, 1329–1337 10.1016/j.jpain.2010.03.006 20542742 PMC2939916

[B94] Peterlin B. L. (2009). The role of the adipocytokines adiponectin and leptin in migraine. J. Am. Osteopath. Assoc. 109, 314–317 19556389

[B95] Peterlin B. L. Bigal M. Tepper S. J. Urakaze M. Sheftell F. D. Rapoport A. M. (2007). Migraine and adiponectin: is there a connection? Cephalalgia 27, 435–446 10.1111/j.1468-2982.2007.01306.x 17448181

[B96] Peterlin B. L. Rosso A. Rapoport A. M. Scher A. I. (2010). Obesity and migraine: the effect of age, gender and adipose tissue distribution. Headache 50, 52–62 10.1111/j.1526-4610.2009.01554.x 19496830 PMC3566428

[B97] Pinhas-Hamiel O. Frumin K. Gabis L. Mazor-Aronovich K. Modan-Moses D. Reichman B. (2008). Headaches in overweight children and adolescents referred to a tertiary-care center in Israel. Obesity 16, 659–663 10.1038/oby.2007.88 18239560

[B98] Pylvänen V. Knip M. Pakarinen A. Kotila M. Turkka J. Isojärvi J. I. (2002). Serum insulin and leptin levels in valproate-associated obesity. Epilepsia 43, 514–517 10.1046/j.1528-1157.2002.31501.x 12027912

[B99] Rainero I. Limone P. Ferrero M. Valfrè W. Pelissetto C. Rubino E. (2005). Insulin sensitivity is impaired in patients with migraine. Cephalalgia 25, 593–597 10.1111/j.1468-2982.2005.00965.x 16033384

[B100] Rains J. C. (2008). Chronic headache and potentially modifiable risk factors: screening and behavioral management of sleep disorders. Headache 48, 32–39 10.1111/j.1526-4610.2008.01119_1.x 18184283

[B101] Rains J. C. Poceta J. (2006). Headache and sleep disorders: review and clinical implications for headache management. Headache 46, 1344–1363 10.1111/j.1526-4610.2006.00578.x 17040332

[B102] Rains J. C. Poceta J. (2010). Sleep and headache. Curr. Treat. Options Neurol. 12, 1–15 10.1007/s11940-009-0056-y 20842485

[B103] Ray L. Lipton R. Zimmerman M. E. Katz M. J. Derby C. A. (2010). Mechanisms of association between obesity and chronic pain in the elderly. Pain 152, 53–59 10.1016/j.pain.2010.08.043 20926190 PMC3004990

[B104] Ray S. T. Kumar R. (2010). Migraine and obesity: cause or effect. Headache 50, 326–327 10.1111/j.1526-4610.2009.01539.x 19804397

[B105] Recober A. Goadsby P. (2010). Calcitonin gene-related peptide: a molecular link between obesity and migraine? Drug News Perspect. 23, 112–117 10.1358/dnp.2010.23.2.1475909 20369076 PMC2947336

[B106] Rigatelli G. Cardaioli P. Dell’avvocata F. Giordan M. Nanjundappa A. Mandapaka S. (2010). May migraine post-patent foramen ovale closure sustain the microembolic genesis of cortical spread depression? Cardiovasc. Revasc. Med. 12, 217–219 10.1016/j.carrev.2010.09.009 21195033

[B107] Robberstad L. Dyb G. Hagen K. Stovner L. J. Holmen T. L. Zwart J. A. (2010). An unfavorable lifestyle and recurrent headaches among adolescents: the HUNT study. Neurology 75, 712–717 10.1212/WNL.0b013e3181eee244 20720191

[B108] Rönnemaa T. Pulkki K. Kaprio J. (2000). Serum soluble tumor necrosis factor-alpha receptor 2 is elevated in obesity but is not related to insulin sensitivity: a study in identical twins discordant for obesity. J. Clin. Endocrinol. Metab. 85, 2728–2732 10.1210/jc.85.8.2728 10946872

[B109] Rozen T. Swidan S. (2007). Elevation of CSF tumor necrosis factor alpha levels in new daily persistent headache and treatment refractory chronic migraine. Headache 47, 1050–1055 10.1111/j.1526-4610.2007.00870.x 17635596

[B110] Scher A. I. Stewart W. Ricci J. A. Lipton R. B. (2003). Factors associated with the onset and remission of chronic daily headache in a population-based study. Pain 106, 81–89 10.1016/S0304-3959(03)00293-8 14581114

[B111] Schütt M. Brinkhoff J. Drenckhan M. Lehnert H. Sommer C. (2010). Weight reducing and metabolic effects of topiramate in patients with migraine – an observational study. Exp. Clin. Endocrinol. Diabetes 118, 449–452 10.1055/s-0030-1248289 20200812

[B112] Seçil Y. Unde C. Beckmann Y. Y. Bozkaya Y. T. Ozerkan F. Basoglu M. (2010). Blood pressure changes in migraine patients before, during and after migraine attacks. Pain Pract. 10, 222–227 10.1111/j.1533-2500.2009.00349.x 20158621

[B113] Sharma A. Pischon T. Hardt S. Kunz I. Luft F. C. (2001). Hypothesis: β-adrenergic receptor blockers and weight gain a systematic analysis. Hypertension 37, 250–254 11230280 10.1161/01.hyp.37.2.250

[B114] Stofkova A. (2009). Leptin and adiponectin: from energy and metabolic dysbalance to inflammation and autoimmunity. Endocr. Regul. 43, 157–168 19908934

[B115] Strelniker Y. M. (2009). Intensive running completely removes a migraine attack. Med. Hypotheses 72, 608 10.1016/j.mehy.2009.01.004 19211193

[B116] Su C. F. Chang Y. Pai H. H. Liu I. M. Lo C. Y. Cheng J. T. (2005). Mediation of beta-endorphin in exercise-induced improvement in insulin resistance in obese Zucker rats. Diabetes Metab. Res. Rev. 21, 175–182 10.1002/dmrr.496 15386812

[B117] Summ O. Charbit A. Andreou A. P. Goadsby P. J. (2010). Modulation of nociceptive transmission with calcitonin gene-related peptide receptor antagonists in the thalamus. Brain 133, 2540–2548 10.1093/brain/awq224 20802202

[B118] Sun-Edelstein C. Mauskop A. (2009). Foods and supplements in the management of migraine headaches. Clin. J. Pain 25, 446–452 10.1097/AJP.0b013e31819a6f65 19454881

[B119] Taylor F. R. (2008). Weight change associated with the use of migraine-preventive medications. Clin. Ther. 30, 1069–1080 10.1016/j.clinthera.2008.06.005 18640463

[B120] Tellez-Zenteno J. Pahwa D. Hernandez-Ronquillo L. Garcia-Ramos G. Velazquez A. (2010). Association between body mass index and migraine. Eur. Neurol. 64, 134–139 10.1159/000316656 20664207

[B121] Tietjen G. E. Peterlin B. Brandes J. L. Hafeez F. Hutchinson S. Martin V. T. (2007). Depression and anxiety: effect on the migraine-obesity relationship. Headache 47, 866–875 10.1111/j.1526-4610.2007.00810.x 17578537

[B122] Tokgoz H. Aydin K. Oran B. Kiyici A. (2012). Plasma leptin, neuropeptide Y, ghrelin, and adiponectin levels and carotid artery intima media thickness in epileptic children treated with valproate. Childs Nerv. Syst. 28, 1049–1053 10.1007/s00381-012-1788-7 22645062

[B123] Touati S. Meziri F. Devaux S. Berthelot A. Touyz R. M. Laurant P. (2010). Exercise reverses metabolic syndrome in high fat diet-induced obese rats. Med. Sci. Sports Exerc. 43, 398–407 20631645 10.1249/MSS.0b013e3181eeb12d

[B124] Trabattoni D. Fabbiocchi F. Montorsi P. Galli S. Teruzzi G. Grancini L. (2011). Sustained long-term benefit of patent foramen ovale closure on migraine. Catheter Cardiovasc. Interv. 77, 570–574 10.1002/ccd.22826 21351231

[B125] Valenzuela R. F. Donoso M. Mellado P. A. Huidobro-Toro J. P. (2000). Migraine, but not subarachnoid hemorrhage, is associated with differentially increased NPY-like immunoreactivity in the CSF. J. Neurol. Sci. 173, 140–146 10.1016/S0022-510X(99)00316-0 10675658

[B126] Van Den Maagdenberg A. M. Terwindt G. Haan J. Frants R. R. Ferrari M. D. (2010). Genetics of headaches. Handb. Clin. Neurol. 97, 85–97 10.1016/S0072-9752(10)97006-1 20816412

[B127] Varkey E. Cider A. Carlsson J. Linde M. (2009). A study to evaluate the feasibility of an aerobic exercise program in patients with migraine. Headache 49, 563–570 10.1111/j.1526-4610.2008.01231.x 18783448

[B128] Varkey E. Hagen K. Zwart J. A. Linde M. (2008). Physical activity and headache: results from the Nord-Trøndelag health study (HUNT). Cephalalgia 28, 1292–1297 10.1111/j.1468-2982.2008.01678.x 18771495

[B129] Viola S. Viola P. Litterio P. Buongarzone M. P. Fiorelli L. (2010). Pathophysiology of migraine attack with prolonged aura revealed by transcranial Doppler and near infrared spectroscopy. Neurol. Sci. 31, S165–S166 10.1007/s10072-010-0318-1 20464613

[B130] Wilmshurst P. T. Nightingale S. Walsh K. P. Morrison W. L. (2005). Clopidogrel reduces migraine with aura after transcatheter closure of persistent foramen ovale and atrial septal defects. Heart 91, 1173–1175 10.1136/hrt.2004.047746 16103551 PMC1769061

[B131] Winter A. C. Berger K. Buring J. E. Kurth T. (2009). Body mass index, migraine, migraine frequency and migraine features in women. Cephalalgia 29, 269–278 10.1111/j.1468-2982.2008.01716.x 19143772 PMC2629138

[B132] Yadav R. K. Kalita J. Misra U. K. (2010). A study of triggers of migraine in India. Pain Med. 11, 44–47 10.1111/j.1526-4637.2009.00725.x 19793343

[B133] Yilmaz I. A. Ozge A. Erdal M. E. Edgünlü T. G. Cakmak S. E. Yalin O. O. (2010). Cytokine polymorphism in patients with migraine: some suggestive clues of migraine and inflammation. Pain Med. 11, 492–497 10.1111/j.1526-4637.2009.00791.x 20113413

[B134] Young W. B. (2008). Prevention treatment of migraine: effect on weight. Curr. Pain Headache Rep. 12, 201–206 10.1007/s11916-008-0035-0 18796270

[B135] Young W. B. Rozen T. (2005). Preventive treatment of migraine: effect on weight. Cephalalgia 25, 1–11 10.1111/j.1468-2982.2004.00819.x 15606563

